# Developing the OpenFlexure Microscope towards medical use: technical and social challenges of developing globally accessible hardware for healthcare

**DOI:** 10.1098/rsta.2023.0257

**Published:** 2024-06-03

**Authors:** Joe Knapper, Freya Whiteford, Daniel Rosen, William Wadsworth, Julian Stirling, Catherine Mkindi, Joram Mduda, Valerian L. Sanga, Paul T. Nyakyi, Thomas Hervé Mboa Nkoudou, Elisée Jafsia, Stephane Fadanka, Kelsey Hummel, Sharmila Anandasabapathy, Richard Bowman

**Affiliations:** ^1^ University of Glasgow, Glasgow, UK; ^2^ Baylor College of Medicine, Houston, Texas, USA; ^3^ Department of Physics, University of Bath, Bath, UK; ^4^ Foxhill Engineering, Bath, UK; ^5^ Ifakara Health Institute, Ifakara, Tanzania; ^6^ Bongo Tech and Research Labs, Dar es Salaam, Tanzania; ^7^ Mboalab and African Higher Institute of Open Science and Hardware (AHIOSH), Yaounde, Cameroon; ^8^ MD Anderson Cancer Centre, Houston, Texas, USA

**Keywords:** global health, open source, pathology, microscopy

## Abstract

The OpenFlexure Microscope is an accessible, three-dimensional-printed robotic microscope, with sufficient image quality to resolve diagnostic features including parasites and cancerous cells. As access to lab-grade microscopes is a major challenge in global healthcare, the OpenFlexure Microscope has been developed to be manufactured, maintained and used in remote environments, supporting point-of-care diagnosis. The steps taken in transforming the hardware and software from an academic prototype towards an accepted medical device include addressing technical and social challenges, and are key for any innovation targeting improved effectiveness in low-resource healthcare.

This article is part of the Theo Murphy meeting issue 'Open, reproducible hardware for microscopy'.

## 1. Introduction

**Figure 1.**
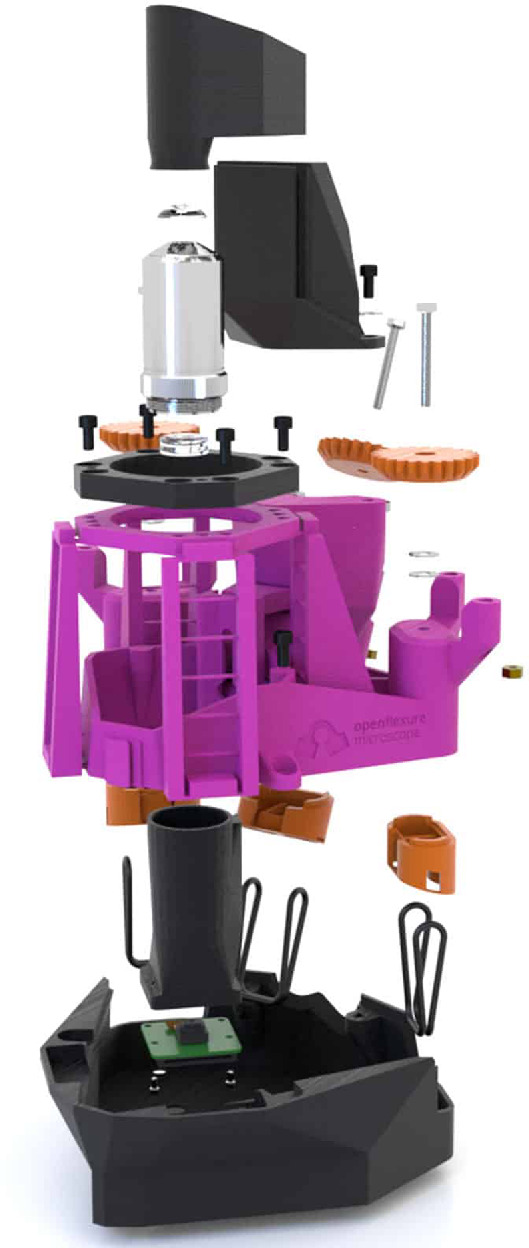
An exploded view of the OpenFlexure Microscope version 6.1.5, reproduced from [1] under the CC-BY-SA licence.

First announced in 2016, the OpenFlexure Microscope is a three-dimensional-printed, accessible microscope capable of automatically moving, focusing and capturing digital images of samples. The hardware (shown in figure 1) and controlling software are open source, meaning users are permitted to manufacture, use, modify and sell the designs without restriction from patents or copyright. This has led to international uptake of the project, with use on every continent for a range of applications. Spin-out projects, including the OpenFlexure Block Stage [2] and OpenFlexure Delta Stage [3], also use three-dimensional-printed monolithic designs to produce high-precision flexure hinges. Other research groups within the rapidly developing community have adapted the OpenFlexure Microscope for imaging modes including super-resolution microscopy, and optical-sectioning microscopy [4,5].

Optical microscopes are a key device in medical diagnostics, used for the identification and characterization of dozens of conditions, including cancer, sexually transmitted infections and parasitic infections [6]. The World Health Organization (WHO) describes manual optical microscopy as the ‘gold standard’ of malaria diagnosis [7]. Malaria is a disease caused by *Plasmodium* parasites in blood, and—despite prognosis being generally positive if diagnosed early—is responsible for over 500 000 deaths per year, with the majority in sub-Saharan Africa. The WHO identifies rapid, accurate diagnoses as the most effective method for reducing the effect of the disease. A blood sample, smeared and stained with a Giemsa stain before manual examination under a light microscope, is the most effective way to diagnose malaria. In addition to the sensitivity and specificity of the method, it can also be performed at the point of care, is versatile (can be used to diagnose other conditions or other sample types) and the cost per test scales favourably.

However, limited access to high-quality, well-maintained microscopes and trained users restricts the accessibility of these tests. A survey of Tanzanian health clinics reported that only 42% had access to a light microscope for malaria diagnosis, dropping to just 20% of public health facilities [8]. Furthermore, there is a worldwide workforce shortage across pathology services [9]. This limited access makes reliable diagnosis less common, and necessitates unreliable procedures. Patients are frequently required to travel to health centres for testing and treatment. Varela *et al*. identify accessibility of reliable transport, the associated financial burden and the duration of the journey as barriers to accessing life-saving treatment [10]. These journeys can involve travelling by car, public transport, bicycles, on foot or some combination thereof, and become less accessible as distance to the healthcare facility increases. The limited accessibility of testing has also led several African countries to operate a ‘hub and spoke’ model for diagnosis, in which samples are collected at local clinics, then transported by motorcycle courier to a central laboratory for testing [11]. This introduces delays and the possibility of sample damage or loss, reducing confidence in local healthcare. This demonstrates the opportunity for improving effectiveness by distributing diagnostic devices to regional health centres.

Improving the accessibility of local diagnosis requires the procurement of high-quality, reliable microscopes. Hardware donations attempt to improve accessibility, but often go unused owing to the need for consumables, maintenance, spare parts and training. We argue that a better solution is the local manufacture, maintenance and use of automated microscopes, not only increasing the availability of devices, but also supporting clinics by allowing the automation of workflow.

This has motivated the development of the OpenFlexure Microscope towards supporting healthcare in low-resource settings. Target environments include the diagnosis of malaria in Tanzania, and oesophageal cancer in Brazil. OpenFlexure Microscope deployment in a teaching hospital in Rwanda is planned in early 2024, with the aim of making diagnosis and education more accessible. While these applications vary in terms of location, sample type and diagnosis procedure, the common challenges are the same. Microscope resolution and contrast must be sufficient to identify the features of interest, and automated sample scanning must be reliable, allowing healthcare workers to perform other manual microscope tasks with confidence. The design and workflow must be reliable and simple, allowing use by non-specialists with a range of backgrounds. Design decisions have been directed by the long-term objective of obtaining medical certification for the OpenFlexure Microscope.

Medical certification is awarded for an assembled product; not underlying design. As such, work is ongoing towards medical certification for sayansiScope, the OpenFlexure Microscope as manufactured in Tanzania by Bongo Tech and Research Labs. This would be the first complete digital diagnostic system manufactured in Tanzania, and would represent a significant step towards local sustainability in healthcare. This aligns with UN Sustainable Development Goals 3, 8, 9 and 12, for Good Health and Well-Being; Decent Work and Economic Growth; Industry, Innovation and Infrastructure; and Responsible Consumption and Production [12].

To maximize uptake and confidence in the OpenFlexure Microscope, we are working to align the design and documentation to the standards required for medical device production. The ISO Standard 13485, ‘Medical devices—Quality management systems—Requirements for regulatory purposes’, is a regulatory standard requiring systematic consideration of the applications, limits and risks associated with a device [13]. This standard covers the full life cycle of a medical device, ‘from the initial conception to final decommissioning and disposal’, and requires documentation of decisions and procedures.

The requirements for becoming medically compliant and suitable for use have been identified and addressed by co-developing the microscope between a range of specialities and locations. Development towards medical use has involved contributions from engineers, physicists, life scientists, computer scientists and healthcare professionals. While the OpenFlexure core team is based in the United Kingdom, the microscope has been co-developed with Tanzanian medical partners and engineering partners from Tanzania, Cameroon and Ghana, with feedback from global health workers based at the Baylor College of Medicine and MD Anderson Cancer Centre, Texas, USA. This has ensured suitability for the end users, based on their requirements and experience.

As the OpenFlexure Microscope continues to be validated in clinical settings, we show how this application has directed the project. We identify steps taken, as well as future work required to ensure utility. These challenges and decisions can broadly be split into technical and social categories, as both are essential for acceptance in healthcare environments.

## 2. Technical challenges and solutions

### (a) Optical performance and automation

The OpenFlexure Microscope is designed to be printed, assembled and used in any environment. The monolithic plastic body can be printed on the lowest quality hobbyist printers, and additional parts have been selected for their reliable supply chains and low cost. A Raspberry Pi computer controls the camera and motors, allowing samples to be scanned automatically. The device can be controlled directly through a mouse and keyboard, through OpenFlexure Connect (a custom-built GUI) or through a range of programming languages including Python and Blockly.

Image-based autofocusing is performed based on the sharpness of the field of view through a range of z positions, measured according to the JPEG file size of the camera stream. Using a 100 × objective magnification, the reduced depth of field necessitates additional steps in autofocusing, using a self-correcting closed-loop approach [14].

The OpenFlexure Microscope supports a range of RMS-threaded objectives, ranging from 0.25 NA to 1.25 NA. Each level of optical power is suitable for different applications and diagnoses, and so components must be selected according to specific use. For the diagnosis of cancer in oesophagus biopsies or pap smears, a 0.25 NA (10 × ) objective is sufficient to give an overview of the sample, while 0.4 NA (20 × ) or 0.65 NA (40 × ) may be required to resolve the finest details. For resolving malaria-causing *Plasmodium* parasites in a blood smear, an oil-immersion 1.25 NA (100 × ) objective is required. The inclusion of index-matching oil and a sub-micron depth of field present additional challenges for automating the imaging of subcellular structures.

While objectives can be swapped on the current OpenFlexure Microscope, this requires removing the sample and unscrewing the objective from within the inverted microscope body. Feedback from pathologists indicates the need for the objectives to be easier to swap, without disturbing the sample position. This has motivated development of an upright microscope design with a swapple objective.

This is achieved through the inclusion of a magnetically constrained, interchangeable three-dimensional-printed mount. There are technical challenges associated with constraining the printed mount sufficiently to achieve a reliabe co-incident positioning of the field of view and the sample. While the inclusion of magnets and other fixings can improve accuracy, we also ensure repeatability by using the camera feed to estimate displacement between objectives; this allows the microscope to automatically correct its own position. The use of camera feedback to improve performance, rather than relying on perfect hardware performance, is a common theme in this project. This self-correcting, closed-loop approach allows high performance without adding to the cost or complexity of the physical device.

### (b) Calibration

Projects with distributed manufacturing are dependent on diverse levels of supply chains, experience and equipment quality to ensure the final product is consistent, regardless of location. For small-scale projects devices can be manually verified; however, this solution scales poorly as the user base grows. This has necessitated the development of a range of microscope self-certification tools. Utilizing the camera to give feedback on image quality, stage mechanics and illumination alignment allows the software to correct for minor differences between devices, while major discrepancies can be identified and corrected before the device is used for patient samples.

**Figure 2.**
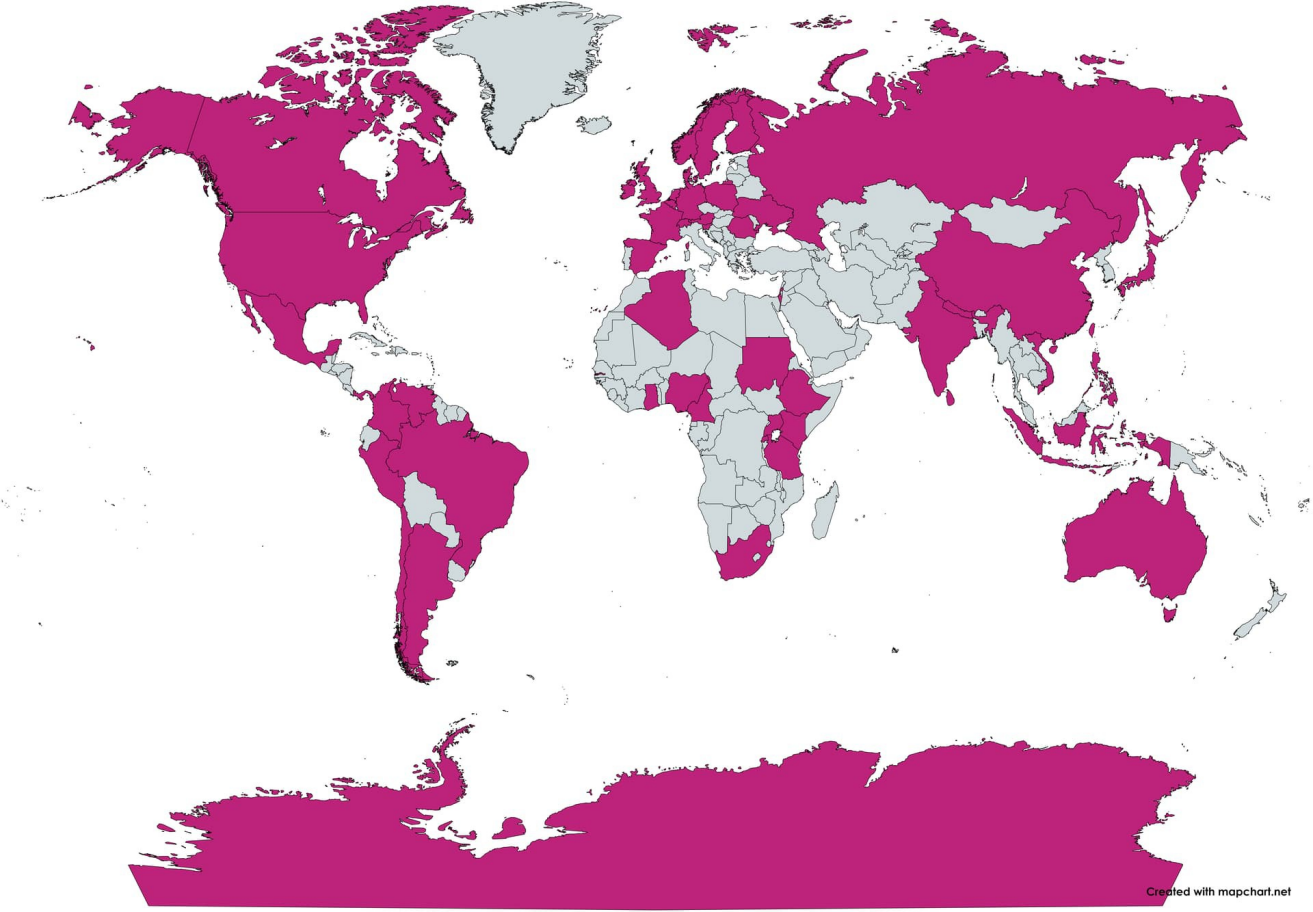
A map of the countries in which the OpenFlexure Microscope has been built or used, according to users in an OpenFlexure forum thread [15].

As shown in figure 2, the OpenFlexure Microscope has been assembled or used in over 45 countries. While a range of microscope calibration procedures are available, they often rely on expensive test targets or specialist interpretation of data [16,17]. To ensure that cost and training do not become additional barriers to entry, OpenFlexure software performs its range of calibration and self-certification procedures on accessible targets.

While calibration of the illumination and camera sensor can be performed without a sample, and stage-to-image calibration can be performed using any sample with obvious features, resolution and physical distance can only be assessed from a target with a known feature size. Our custom calibration software (MICAT) [18] uses a USAF 1951 resolution target (approximate cost £200, from a range of suppliers), which has a pattern of decreasingly spaced periodic grates. Determining the pixel size from images of these features of known size allows calibration between motor position, image coordinates and physical sample location. Capturing images of a large slanted edge allows estimation of the line spread function (a one-dimensional analogue to the point spread function). This resolution metric can identify a poorly assembled microscope, advising the user that the resolution limit may be higher than the features they need to examine.

In the case that these calibration steps indicate insufficient performance, the software can advise users of possible causes and suggest next steps, including returning the microscope to the manufacturer for maintenance. As a low-cost, modular product, the system can be repaired or replaced locally, aligning with the ISO 13485 standard requirement of considering the end of life of a medical device. This is in contrast with many proprietary systems donated from the ‘Global North’, which can restrict maintenance and repairs.

We suggest a workflow in which each newly assembled device is fully calibrated, with the most essential characteristics also being automatically re-tested at the start of each workday. The results of each calibration run are archived, allowing full traceability between certification and medical scans. This would be part of the basis of a risk-management procedure, required for medical devices by ISO 14971, ‘Medical devices—Application of risk management to medical devices’ [19]. This would ensure that issues are identified and the user informed before they can affect patient outcomes.

While not critical for diagnosis, other performance-improving monitoring of the microscope can be carried out during ongoing scans. The motion of the translation stage of the OpenFlexure Microscope follows a sphere-cap, and so the parasitic motion in z for an xy move can be predicted. Therefore, a focus map of the sample can speed up image acquisition. Measuring how long the stage takes to settle after movements allows images to be collected as soon as the system is stable. More accurate movement and indentification of the limits of the range of motion can be achieved by performing closed-loop movements; using the camera stream to feedback on the success of each movement. While these steps cannot replace the knowledge of experienced users, they allow new users to have confidence in the device performance.

## 3. Organization of a distributed project

Managing and directing a distributed, open project requires active collaboration and networking; the publication of source code and designs is only the first step in making the project accessible. How to best manage a collaborative project is both a technical and social problem, as users require both technical skills and motivation to share their work. Any barrier to the free sharing of knowledge and experience can decrease the effectiveness of a project.

### (a) Capturing an evolving design

Documentation of the design, decisions and knowledge of a project is key to OpenFlexure development for two reasons. First, medical certification requires an archive of decisions, allowing the project history to be traced. Second, as an open-source project, designs are released in the hope that users can build, use and modify the design without requiring direct contact with the original development team.

Capturing the decisions and discussions of a multinational evolving team is non-trivial, and requires tools developed for supporting collaboration. OpenFlexure version control is performed in Git, a distributed system primarily used for software development. While GitLab hosts the OpenFlexure software, including the server, GUI and calibration procedures, it also hosts the microscope CAD files, acting as a version control system for an evolving hardware design.

While GitLab—as a public forum for discussion—is preferred by the core development team for its archiving, traceability and openness, feedback from some collaborators suggests a preference for communication through private channels such as WhatsApp or email. Whether due to protecting intellectual property, being unwilling to publicly share suggestions or preferring the wider use of these platforms, this moves some discussion and decisions into private channels. The project will often benefit from the core development team publicly recording the outcomes of these conversations at a later date. Otherwise, there is significant risk of replicating previous work, discussion or mistakes.

The multiple variants of the OpenFlexure Microscope, customized to meet a range of budgets, applications and users, make documenting the designs more challenging. GitBuilding was developed as a platform for documenting and tracking scientific hardware builds [20]. Originally written to meet the needs of the OpenFlexure Microscope, GitBuilding has been adopted by multiple projects as part of the Gathering for Open Science Hardware (GOSH) [21].

Key features of GitBuilding, which benefit the OpenFlexure project, include automatically tracking the parts count to generate a bill of materials, and allowing multiple projects with shared steps to link the relevant pages. For the OpenFlexure Microscope, this means that pages which are common to multiple versions need only be written or updated once, and all dependent documentation will automatically update. These ease-of-use improvements in documenting an evolving design are essential to encourage projects to keep their instructions and designs properly co-ordinated.

### (b) Diverse priorities

The use of medical devices can change based on application and environment. In addition to various samples requiring different magnification or imaging modes, the broader workflow and priorities of each clinic vary. Priorities can only be balanced on a site-by-site basis, and with first-hand experience in the relevant environment. Examples of these issues include balancing speed with reliability, customization with known performance and accessibility with quality.

The most reliable scans will run slowly, with large overlaps between regions and frequent refocusing. While imposing delays on the results of a scan, the consequences of a failed scan delay results and disrupts the workflow, reducing confidence in both the device and the healthcare system. While work is ongoing to enable a narrow set of diagnostic tests (primarily malaria diagnosis in blood samples), light microscopy remains a versatile method for diagnosis. To maximize the potential effect of the OpenFlexure Microscope in global healthcare, the long-term objective must be as a generic laboratory device. However, in the short term, limiting scope increases the value of feedback and allows developers to focus their efforts to set procedures.

Accessibility is vital to the effect of the OpenFlexure Microscope; the supply chain, documentation and software must all lower barriers to entry. While higher resolution images would be achievable with higher-performance components, the increased cost and restricted supply chains would decrease the overall effect of the project. The feedback of clinicians helps inform the development team where the balance lies, and will depend on the resources at each site.

While the development team can adjust and advise according to each environment, the ultimate goal for an open project is for users to adopt the technology without direct support from the core team. As such, decisions, options and conversations should be archived publicly, distributing the project and allowing potential users to make informed choices based on their priorities. Our public forum invites the community to post challenges, options and suggestions with the goal of both technology education and dissemination.

## 4. Social obstacles and opportunities

Uptake of new technology in diverse healthcare environments necessitates acceptance by local healthcare workers. We have found that engaging with target environments early and actively is important to ensure that proposed solutions are engaged with. As we continue testing and validation of the OpenFlexure Microscope in varied global clinical settings, receiving stakeholder feedback will be critical to acceptance and dissemination of the technology. Indeed, we will initiate usability and feasibility testing in these settings to determine barriers and facilitators to dissemination.

### (a) Clinician trust

Ensuring the trust of clinicians is key to all future goals for microscope deployment in healthcare. Testing the device, receiving relevant feedback and ultimate deployment in healthcare systems requires the time and support of local clinicians. Methods of encouraging the necessary confidence is a complex issue, and varies between specialities and regions. Although medical certification would give credence to the OpenFlexure Microscope, feedback from target end users indicates that this is only one possible avenue to acceptance.

Local manufacture of hardware would represent a shift in medical device procurement in many of the target environments. Such a fundamental change in supply chain is expected to divide user opinion, depending on their experience of previous devices and local engineering. While the OpenFlexure Microscope is designed to be built and maintained by users with minimal training, early feedback from clinicians suggests they would only use a device assembled by an external supplier, and would return it to them for any maintenance. Whether users prefer local or international provenance needs to be captured by region, informing how the OpenFlexure Microscope should be presented in each area.

**Figure 3.**
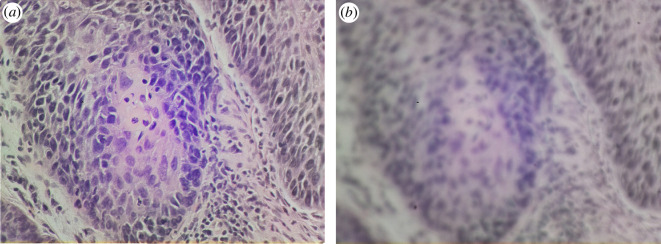
Images of a squamous cell carcinoma from a vocal cord sample, captured on the OpenFlexure Microscope with a 40 × objective. Panel (*a*) shows a focused image with well-aligned optics, while panel (*b*) shows the same area out of focus and with misaligned illumination. A trained user can distinguish between these images, refusing to give a diagnosis based on poor data.

Clinicians also distinguish between the use of a microscope and other diagnostic devices such as a pulse oximeter. A pulse oximeter tests the blood oxygen levels of the user, and displays a number to the clinician. In the absence of contradictory symptoms, this value represents the only believable metric, making frequent re-certification of the device a necessity. In contrast a microscope displays images to an experienced user, allowing for a decision based on device performance. Clinicians can then judge whether an image is of sufficient quality to make a diagnosis, or whether device maintenance is required (as in figure 3). While poorly performing devices would still delay test results, they present less of a risk to patients than so-called ‘black box’ devices.

A medical device needs to be more reliable than equipment in many other settings, especially in limited-resource environments. Failed scans can reduce clinician faith in the project, while also wasting the time and resources of both the workers and patients. Academic projects with medical applications also need to consider the most suitable point to begin contact with healthcare workers. Asking for feedback too early can waste clinician time, as the project direction may be too vague for useful assessment. Including medical experts too late can instead waste developer time, causing misidentification of challenges or the proposal of unsuitable solutions. The OpenFlexure project has medical collaborators from a range of backgrounds and locations, allowing regular feedback sessions to ensure priorities remain aligned.

The lack of accessibility to microscopes is also a key limitation in education. Optical microscopes are essential tools in life-science research and healthcare, but can be prohibitively expensive or difficult to source, limiting hands-on training. Allowing students the opportunity to build, maintain and use their own microscope would improve their technical skills and understanding of their equipment. The OpenFlexure Microscope is especially suitable for education, as digital images can be annotated, shared and archived; in addition, the ambiguity of where to look on a sample when training medics on eyepiece-based microscopes is removed. The long-term objective of introducing the OpenFlexure Microscope into the curriculum would strengthen trust from health workers, and is reliant on high-capacity local manufacturing. However, presentation should be considered carefully; it is essential to not give the impression that the OpenFlexure Microscope is ‘only’ suitable for students or children, but is, rather, a versatile scientific device.

### (b) Ease of use

While the technical performance of a device is important for its uptake, it also needs to be suitable for untrained users. Workflows relying on knowledge of Git, Jupyter and Linux are suitable for development, but present a significant barrier to entry for clinicians and end users. While development of the user experience may not directly contribute to publications or other academic priorities, increased uptake can greatly increase collaboration.

OpenFlexure Connect is released as open software to provide an intuitive GUI for controlling OpenFlexure devices. It is intended for the majority of users, supporting the most common functions such as capturing images, autofocusing and scanning samples. Necessary calibration steps appear in a wizard, and new users have the option of a guided tour of the functionality. Combined with strong branding and engineering efforts to improve the appearance of the hardware, the first impression of the OpenFlexure system is intended to inspire confidence, appearing as a product rather than a prototype.

Balancing user experience with the need for user control is especially challenging when some users require further in-depth knowledge of software settings, while other locations want to prevent users from adjusting the defaults. For medically certified use, the software must only be used in the approved state, without the additional plugins or extensions normally available in OpenFlexure software. In OpenFlexure Connect, running in blood-sample scanning mode adds additional fields such as a patient identifier, and locks the size and shape of the scan to the accepted standard. When deployed in clinical settings, it will also disable additional extensions and plugins, ensuring that only approved code is used.

For our automated sample scanning, we set default thresholds based on the appearance of H&E stained biopsies. While this covers the majority of current applications, future users might work with other stains, which affect the appearance of the sample. While adjusting the thresholds in the software requires only a minor change, of more challenge is the design of a GUI such that users only need to see these settings when required. The accessibility of the project is also greatly increased by the contribution of translation into additional languages, including French and Spanish [22,23].

## 5. Conclusion

We have described the considerations and challenges involved in turning an academic prototype into a medical device for global health. Some of these considerations are specific for microscopy, while others apply generally to improving the accessibility of healthcare. This development extends beyond purely technical challenges; the project requires acceptance from clinicians to test, feedback on and use the device.

This development has surpassed the typical scope of an academic project—the further release of manufacturing-ready designs, technical documentation and support are all required for widespread adoption. We argue that, although this development is rarely strategic for academic researchers, it benefits from the involvement of the original core team [24].

The OpenFlexure Microscope is currently at Technology Readiness Level 7; it has been tested in operational environments, scanning patient blood samples in Tanzanian health clinics and biopsies at the Michael E. DeBakey VA Medical Center in Texas. Continuing development to align with ISO 13485 gives the technology the greatest chance for widespread effectiveness. The opportunity to support healthcare and education directs OpenFlexure development, with potential users providing a ‘demand pull’ as well as ‘technology push’. The project benefits from a distributed network of partners, with specialist knowledge contributed by Mboalab, Bongo Tech and the Baylor College of Medicine. We have shown that the effectiveness of implementation of this project cannot be achieved by technical performance alone, but also requires collaboration and co-development with all stakeholders.

## Data Availability

This article has no additional data.
